# Wheelchair Control in a Virtual Environment by Healthy Participants Using a P300-BCI Based on Tactile Stimulation: Training Effects and Usability

**DOI:** 10.3389/fnhum.2020.00265

**Published:** 2020-07-10

**Authors:** Matthias Eidel, Andrea Kübler

**Affiliations:** Institute of Psychology, University of Würzburg, Würzburg, Germany

**Keywords:** brain-computer interface (BCI), event-related-potential (ERP), P300, tactile, wheelchair control, tactually evoked potentials, replication

## Abstract

Tactile stimulation is less frequently used than visual for brain-computer interface (BCI) control, partly because of limitations in speed and accuracy. Non-visual BCI paradigms, however, may be required for patients who struggle with vision dependent BCIs because of a loss of gaze control. With the present study, we attempted to replicate earlier results by Herweg et al. (2016), with several minor adjustments and a focus on training effects and usability. We invited 16 healthy participants and trained them with a 4-class tactile P300-based BCI in five sessions. Their main task was to navigate a virtual wheelchair through a 3D apartment using the BCI. We found significant training effects on information transfer rate (ITR), which increased from a mean of 3.10–9.50 bits/min. Further, both online and offline accuracies significantly increased with training from 65% to 86% and 70% to 95%, respectively. We found only a descriptive increase of P300 amplitudes at Fz and Cz with training. Furthermore, we report subjective data from questionnaires, which indicated a relatively high workload and moderate to high satisfaction. Although our participants have not achieved the same high performance as in the Herweg et al. (2016) study, we provide evidence for training effects on performance with a tactile BCI and confirm the feasibility of the paradigm.

## Introduction

By establishing a direct link between brain and computer, brain-computer interfaces (BCI) allow their users to communicate and interact with the environment. The electrical activity of the brain can be measured non-invasively *via* electroencephalography (EEG) and interpreted by a computer. Many BCI paradigms rely on event-related potentials (ERP), such as the P300 (Squires et al., 1975; Polich and Margala, 1997). The P300 can be reliably elicited by focusing attention on rare stimuli (targets) while ignoring other, frequent stimuli (non-targets), and occurs as a positive deflection about 300 ms post-stimulus. It is usually measured at electrode positions Fz, Cz, and Pz (Polich, 1986).

Because of their independence from voluntary muscular function, BCIs are a promising tool to assist severely paralyzed patients, for example, those with amyotrophic lateral sclerosis (ALS), brain injury, or one of many other causes for motor impairment (Vidal, 1973; Wolpaw et al., 2002). These potential end-users, however, may face usability issues with visual BCIs once their vision or gaze control becomes impaired (Birbaumer and Cohen, 2007; Brunner et al., 2010). Hence, vision-independent BCI paradigms which rely on ERP elicitation *via* auditory and tactile stimulation have been developed and demonstrated to be viable (Schreuder et al., 2011; Furdea et al., 2009; Brouwer and van Erp, 2010; Hill and Schölkopf, 2012; Riccio et al., 2012; Kaufmann et al., 2014; Simon et al., 2015; Baykara et al., 2016; Halder et al., 2016).

Table 1 provides an overview of P300 BCI studies using tactile paradigms along with their mean accuracies (i.e., percentage of correct classifications), information transfer rates (ITR) that were achieved, and the number of possible selections (classes), including distractors. Examples include a tactile spelling BCI by van der Waal et al. (2012) which used small taps on the fingertips, administered by a braille stimulator, to achieve a mean accuracy of 67% among a sample of twelve healthy participants, and one of the earliest publications, where up to six vibrotactile devices were located around the waist of healthy participants, achieving a 58% mean accuracy (73% for two devices; Brouwer and van Erp, 2010). Notably, several studies demonstrated that the tactile modality can be feasible for BCI control by severely impaired potential end-users (Lugo et al., 2014; Ortner et al., 2014; Severens et al., 2014; Guger et al., 2018).

**Table 1:** Overview of recent tactile P300 brain-computer interface (BCI) studies.

Study	Mean accuracy (%)	Mean ITR (bits/min)	Classes	Population	Analysis
Brouwer and van Erp (2010)	68^a^	3.71	6	Healthy	Online
van der Waal et al. (2012)	67.0	7.8	36	Healthy	Offline
Thurlings et al. (2012a)	78^a^	6.52	6	Healthy	Offline
Ortner et al. (2012)	68.1	3.36	8	Healthy	Online
Severens et al. (2013)	77.0	1.2	2	Healthy	Online
Severens et al. (2014)	60.0	6.6	36	Healthy	Offline
Severens et al. (2014)	58.0	6.6	36	ALS	Offline
Kaufmann et al. (2014)	85.8	2.54	4	Healthy	Online
Herweg et al. (2016)	95.6	20.73	4	Healthy	Online
Halder et al. (2018)	71.0	3.4	5	Healthy	Offline
Chabuda et al. (2019)	76	0.51	2	Healthy	Online

*Notes: a = approximation. When multiple variations of a paradigm were reported, those with the highest ITRs are included in this table. If numerical values were not reported, we provide approximations based on plots. Hybrid BCIs that used a combination of modalities are not included*.

Furthermore, a tactile P300 paradigm specifically intended for wheelchair control was developed and tested with healthy participants aged 50–73 years (Kaufmann et al., 2014; Herweg et al., 2016). Here, vibrotactile stimulation was applied at body positions roughly corresponding to the four selectable movement commands (front, back, left, and right). Importantly, the study by Herweg et al. (2016) had each participant attend five sessions, throughout which significant effects of training on the P300 amplitudes could be demonstrated. No significant increase could be shown for online BCI performance measures (accuracy and ITR), a fact that the authors attributed to a ceiling effect due to very high performances already during the first session. Similar training effects had been reported for auditory paradigms before (Käthner et al., 2013; Halder et al., 2016). However, Herweg et al. (2016) introduced an additional BCI task with individually optimized conditions during the fifth session. Here, the number of sequences was reduced to an average of 2.25 (approx. 5.6 s per command), depending on the participants’ individual performances. With the thus shortened stimulation phase, a mean BCI accuracy of 95.56% and a mean ITR of 20.73 bits/min were achieved. To our knowledge, these values are still the highest among comparable paradigms (see Table 1). Considering the advanced age of the participants, and the fact that mechanoreception is known to become less sensitive over the years (Cauna, 1964; Iwasaki et al., 2003; Wickremaratchi and Llewelyn, 2006), these results were rather encouraging and motivated further research. Particularly, we were interested in replicating the surprisingly high performances. Therefore, the current study implements the same general experimental design to further solidify the evidence for training effects and the general feasibility of the paradigm.

We hypothesized that physiological measures (P300 amplitudes and differences between curves, H1) and BCI efficiency (ITR, H2) would increase with training across five sessions. Furthermore, we expected our participants to be able to navigate the wheelchair with at least 70% accuracy in their last session (H3). Because of the previous excellent performance of elderly participants (Herweg et al., 2016), we expected that participants would not be negatively affected by advanced age (as compared to young adult users, H4). Additionally, we added questionnaires about usability, namely workload and general satisfaction as suggested in the framework of the user-centered design approach (for a review of user-centered BCI design, see Kübler et al., 2014).

## Materials and Methods

### Participants

*N* = 16 healthy participants were recruited. One subject wished to terminate participation after the third session due to incompatibilities with their schedule, leaving 15 participants for analysis (three male, age range 20–61 years, *M* = 38 years, *SD* = 15.4). All reported (corrected to) normal vision and were naïve concerning BCI operation. They either received a monetary reimbursement of € 7.50 per hour, or, in the case of local psychology students, course credits. All gave informed consent to the procedure, which was approved by the ethical review board of the Institute of Psychology at the University of Würzburg, Germany (GZEK 2013-11).

### EEG Recording and Processing

EEG was recorded with a sampling rate of 512 Hz with 12 passive Ag/AgCl electrodes and amplified using a g.USBamp (g.tec Engineering GmbH, Graz, Austria). Electrode positions were Fz, FC1, FC2, C3, Cz, C4, P3, Pz, P4, O1, Oz, and O2 (Sharbrough, 1991), with ground and reference electrodes at the right and left mastoids, respectively. Impedances were kept below 5 kΩ. Online filtering included a bandpass filter between 0.1 and 60 Hz and a notch filter between 48 and 52 Hz.

For offline analysis, EEG data was bandpass filtered between 0.1 and 30 Hz and divided into segments of 800 ms post-stimulus, plus 100 ms pre-stimulus for baseline-correction. Segments containing values exceeding a threshold of ±150 μV were excluded. Target and non-target epochs were averaged separately. Data were analyzed with MATLAB© (v2013b) using adapted scripts provided by BCI2000 (Schalk et al., 2004) and EEGLab (Delorme and Makeig, 2004). Classifier weights were defined using the stepwise linear discriminant analysis (SWLDA) as implemented in the BCI2000 package.

### Stimulation

**Figure 1:**
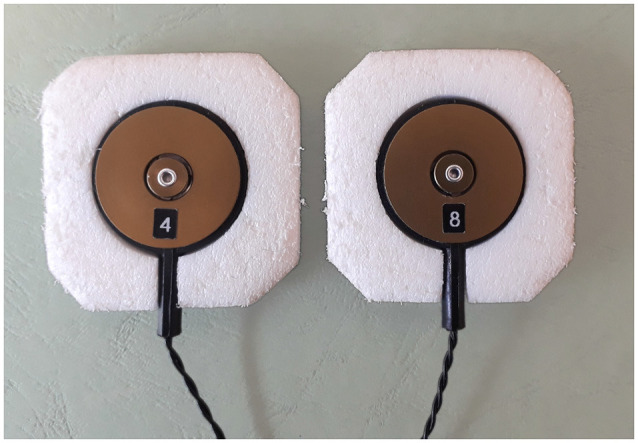
Two tactile actuators from the C2 tactor system by Engineering Acoustic Inc., Casselberry, FL, USA.

As in the original study by Herweg et al. (2016), tactile stimulation was applied at right and left thigh (next to the knee), abdomen (1–5 cm above the navel) and neck (at the height of the C4–Th3 dermatomes) *via* a BCI2000-controlled tactor device (C2 tactors; Engineering Acoustic Inc., Casselberry, FL, USA; see Figure 1). These body positions were chosen to be easily associable with the desired direction, since such congruence of directions (e.g., left thigh encoding the left turn command) may be beneficial for BCI performance (Thurlings et al., 2012b). Two tactile actuators were used per position to ensure well perceivable stimulation. The devices were adjusted until they were reported as equally strong for all positions. During the BCI session, the four tactor positions were activated (vibrating at 250 Hz for 220 ms) in a pseudorandomized sequence with equal probabilities (25%). The interstimulus interval was 400 ms.

### Procedure

**Figure 2:**
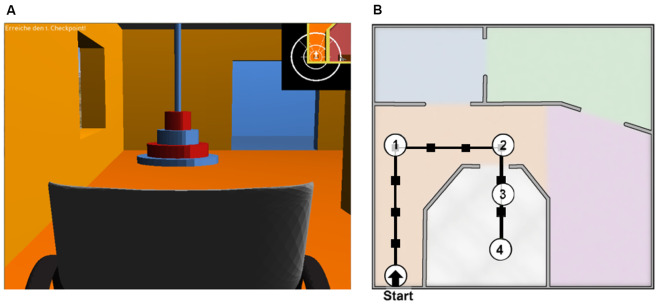
Overview of the virtual environment from the navigation task. **(A)** User’s perspective, showing the wheelchair and a checkpoint in front of it. **(B)** Floorplan of the apartment showing one of the courses. Adapted from Kaufmann et al. (2014).

Participants were sitting in a chair in front of a desk, on which a monitor showed the virtual environment for wheelchair navigation from the perspective of a wheelchair user (however, as visible in Figure 2A, the backrest of the wheelchair was shown to better allow for an estimation of its dimensions). They were instructed to keep their eyes open and to avoid blinking, unnecessary movements and to keep facial muscles relaxed during recording.

To be able to investigate the training effects of repeated BCI use, participants were invited for five sessions on separate days, with no more than one week between sessions. At the beginning of each session, three calibration runs were performed. Participants had to concentrate on the stimuli of a target body position which was indicated *via* text on the monitor. Other stimulations (non-targets) had to be ignored. Overall, this resulted in a total of 240 target and 720 non-target trials per participant.

Every session, the participants’ current calibration data served to train an SWLDA classifier (Schalk et al., 2004), which was then used for two navigation runs, For this task, participants had to navigate a wheelchair through a virtual 3D apartment (shown on the monitor, see Figure 2A) consisting of four interconnected rooms and an L-shaped hallway (Kaufmann et al., 2014; Herweg et al., 2016). The current course was defined by four checkpoints that had to be reached in a fixed order (see Figure 2B). Navigation was semi-free, meaning that the path was only guided by checkpoints, but the track to the checkpoints was not preset. Thus, the users first had to choose a direction and then focus on the stimuli applied to the corresponding body position (i.e., on the left thigh to make a 45° left turn). Start and endpoints of the course were switched after every run, such that participants had to navigate back to the original starting point. Each complete run required at least 14 commands; however, all erroneous or misleading commands were executed (except those leading to collisions, which were counted but interrupted) and had to be either corrected or incorporated into an alternative route toward the checkpoint. As established in the study by Herweg et al. (2016), the run was terminated if the final checkpoint was not reached after a maximum number of 22 commands. To preclude the putative ceiling effect of this previous study, which used a fixed number of eight sequences for one command, we adjusted this number individually based on the calibration runs. The number of sequences predicted for 100% accuracy was chosen, up to a maximum of 10 sequences.

At the end of each session, participants filled in the NASA-TLX (Hart and Staveland, 1988) to assess workload, and a bipolar 11-point visual analog scale (VAS) for satisfaction with BCI control, ranging from “very frustrated” to “very satisfied.” At the end of sessions one and five, participants filled in the TUEBS (Zickler et al., 2011), measuring satisfaction with an Assistive Technology Device. The TUEBS is based on QUEST 2.0 and adapted to BCI (Demers et al., 2002; Kübler et al., 2014).

### Statistical Analysis

We chose the ITR as the primary measure for performance because accuracy was expected to remain mostly constant due to individually adapting the number of sequences. The ITR is the amount of information transferred during a given time in bits per minute. The number of bits (B) is calculated with the following equation, using the accuracy (P) and the number of all possible selections (here, *N* = 4):

$$B=log_{2}N+Plog_{2}P+(1-P)log_{2}\frac{\left(1-P\right)}{\left(N-1\right)}$$

The ITR is then calculated by multiplying the number of bits with the number of selections per minute. Therefore, the resulting value is directly dependent on the number of possible selections, the number of sequences (and thus, the time required for one command), and the actual online accuracy. In the present paradigm, the number of sequences was adapted dynamically, thus, stimulation time varied. For instance, assuming a number eight sequences (as in Herweg et al., 2016), one command could be given every 19.8 s (3.0 selections per minute). Additionally, we calculated average offline accuracies from the calibration run data based on a leave-one-out cross-validation procedure.

We extracted the physiological ERP features mean target amplitudes and mean difference between target and non-target from averaged EEG data in the time window of 300–500 ms post-stimulus at positions Fz, Cz, and Pz.

To test for training effects, BCI performance measures, physiological features, and questionnaire data from all sessions entered analysis (performed with IBM SPSS 25^®^). Because of small sample sizes and violations of assumptions for ANOVA, Friedman ANOVAs were calculated unless mentioned otherwise.

## Results

Fifteen participants completed all five sessions of the study, resulting in 75 full BCI sessions available for analysis. Nine participants reached BCI efficiency, but participants 6, 7, 8, 10, 11, and 16 did not (efficiency was assumed when the mean online accuracies ≥70% were reached during the last session, or in at least three other sessions). To adequately describe training effects, only BCI efficient participants entered the respective analyses (i.e., all statistics, tables, and figures). The questionnaire analysis was considered relevant for all 15 participants and thus performed on the full sample.

### Physiological Measures

**Figure 3:**
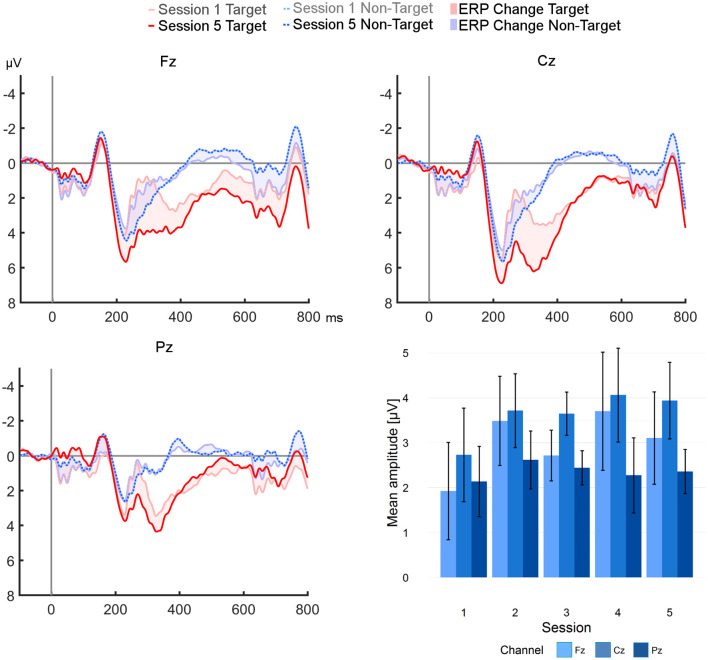
Comparison of session 1 vs. 5 grand averages of target and non-target epochs to 800 ms post-stimulus. The highest target peak (6.21 μV) after training was observed at position Cz. The bar plot shows mean amplitudes from all sessions (error bars represent SE).

**Figure 4:**
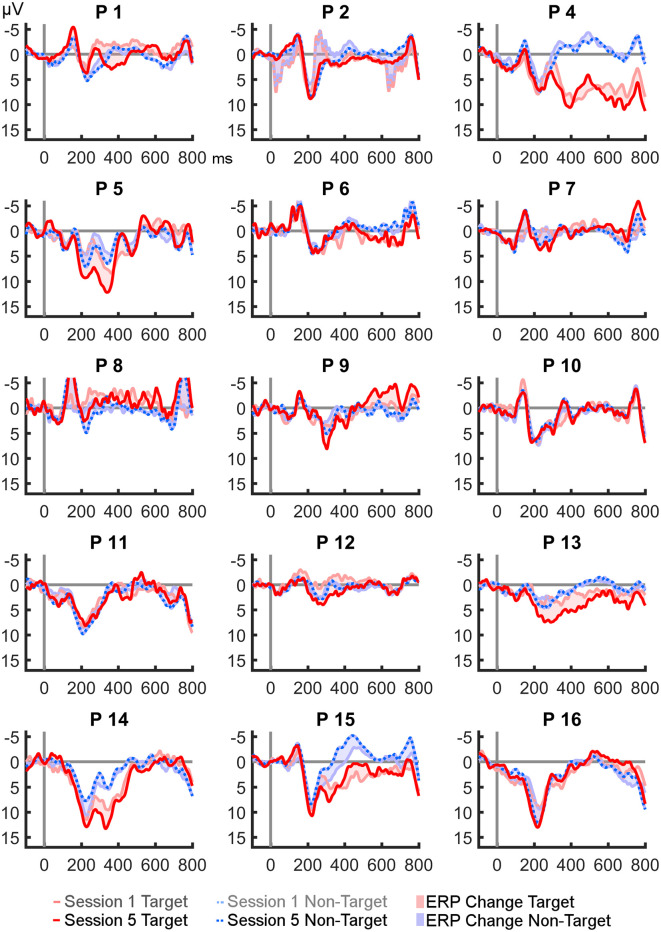
Comparison of target and non-target epochs at Cz from session 1 vs. 5, per participant.

Grand averages of target and non-target curves at Fz, Cz, and Pz from session one and five, and the development of ERP amplitudes across all sessions are plotted in Figure 3. Descriptively, both amplitudes and differences between the curves increased from the first to the last session in the P300 range at Fz and Cz. However, Friedman ANOVAs did not reveal significant effects on amplitudes or differences throughout the study (see Table 2 for an overview of descriptive statistics and Friedman ANOVAs). Epochs from position Cz (where amplitudes were highest) are plotted in Figure 4 for every participant. Visual analysis revealed a high degree of heterogeneity between participants.

**Table 2:** Statistics for event-related potentials (ERP) measures at Fz, Cz and Pz.

Electrode position		Fz	Cz	Pz
Amplitudes	Mean S1 (SD; μV)	1.92 (3.26)	2.73 (3.13)	2.14 (2.35)
	Mean S5 (SD; μV)	3.10 (3.08)	3.94 (2.56)	2.36 (1.48)
	Chi-square (*χ*^2^)	1.96	5.33	0.44
	Significance (*p*)	0.769	0.261	0.985
Difference between curves	Mean S1 (SD; μV)	1.48 (4.07)	2.32 (3.30)	2.14 (2.26)
	Mean S5 (SD; μV)	2.70 (4.05)	3.50 (2.70)	2.38 (1.77)
	Chi-square (*χ*^2^)	5.96	2.04	1.51
	Significance (*p*)	0.210	0.754	0.848

*No significant increases were supported by Friedman ANOVAs*.

### BCI Performance

**Figure 5:**
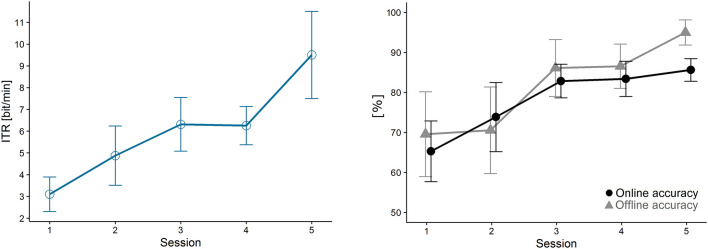
Online brain-computer interface (BCI) performance from the navigation tasks. Data were averaged over all BCI efficient participants (error bars represent SE). Both information transfer rate (ITR; *p* = 0.002), online accuracy (*p* < 0.05) and offline accuracy (*p* < 0.01) increased significantly with training.

The number of sequences was adjusted as explained in the methods section. As a result, participants received an average number of *M* = 6.7 (16.6 s per command) sequences in the first, and *M* = 3.8 (9.4 s per command) in the last session. Figure 5 shows the development of BCI performance (ITR and accuracies) over the five training sessions: Mean online ITRs increased significantly from 3.10 to 9.50 bits/min (Friedman, *p* < 0.01). Similarly, mean online accuracies increased from 65% to 86% (*p* < 0.05). All online performances are summarized in Table 4 to illustrate the heterogeneity of results. Cross validated offline accuracies were calculated based on calibration data using the maximum number of (10) sequences available for classification. Offline accuracies increased highly significantly between sessions, from 70% to 95% (*p* ≤ 0.01). These values greatly exceeded the chance level threshold of approximately 40% (using an alpha of 0.05) to be considered non-random and statistically significant (Müller-Putz et al., 2008). Table 3 summarizes results from all Friedman ANOVAs.

**Table 3:** Mean values of BCI performance measures from sessions one and five with results from Friedman ANOVAs (calculated over all sessions).

Measures	Online accuracy	Offline accuracy	Online ITR (bits/min)
Session 1 (SD)	65% (23.79)	70% (31.86)	3.10 (2.37)
Session 5 (SD)	86% (8.38)	95% (9.43)	9.50 (6.01)
Chi-square $\chi_{\left(4,N=9\right)}^{2}$	9.27	12.35	15.29
Significance (*p*)	0.048*	0.010*	0.002*

*Asterisks indicate significant effects*.

**Table 4:** Individual online performances per participant and session.

	Online accuracy (%)					Online ITR (bits/min)				
Participant	S1	S2	S3	S4	S5	S1	S2	S3	S4	S5
1	70.5	96.7	59.1	75.0	75.0	2.65	4.67	2.27	4.79	3.83
2	88.9	80.5	77.8	93.8	83.3	5.33	3.38	5.34	6.31	5.25
4	93.8	96.7	91.2	93.5	96.7	6.31	10.50	11.53	9.39	21.00
5	63.6	86.8	96.7	89.5	93.8	2.89	3.72	8.40	8.15	12.61
6^ex^	75.0	29.5	56.8	63.6	52.3	2.74	0.02	0.80	1.16	0.59
7^ex^	45.5	38.6	29.5	36.4	43.2	0.34	0.17	0.02	0.11	0.39
8^ex^	43.2	31.8	27.3	36.4	38.6	0.27	0.04	0.00	0.11	0.16
9	22.7	72.5	77.5	73.7	83.3	0.00	2.89	3.52	4.54	5.25
10^ex^	47.7	38.6	25.0	45.5	34.1	0.42	0.16	0.00	0.34	0.09
11^ex^	52.3	31.8	34.1	27.3	38.6	0.59	0.05	0.07	0.00	0.16
12	40.9	22.7	70.5	56.8	72.7	0.21	0.00	3.17	1.33	3.50
13	56.8	38.6	91.7	83.7	82.9	0.80	0.16	5.03	5.33	8.63
14	81.8	91.2	86.8	100.0	88.9	4.97	11.53	4.96	9.68	15.98
15	68.2	77.5	93.8	84.2	93.8	4.78	7.05	12.61	6.78	9.46
16^ex^	68.2	66.7	77.5	65.9	59.1	1.44	2.23	3.02	2.58	1.51

*Notes: ^ex^ = excluded, S = session*.

### Effects of Age

There was a notable difference in performances depending on age. To test hypothesis H4, we split participants into two groups, young adults (YA, ages 20–33, *N* = 8) and middle-aged (MA, ages 41–61, *N* = 7). Four of six cases of BCI inefficiency occurred among the MA group, and a Spearman test (without exclusion of inefficient cases) revealed a strong negative correlation between age and mean ITR (*r*_s_ = −0.69, *p* < 0.01). Due to the hence drastically reduced sample size in either group (YA = 6; MA = 3), we report descriptive ITR values only (see Table 5). Online ITRs of both groups increased substantially with training, but the YA group strongly outperformed the MA group.

**Table 5:** Information transfer rate (ITR) values from sessions one and five, split by age group.

Mean values	YA	MA
Session 1 ITR (SD; bits/min)	4.49 (1.43)	0.34 (0.41)
Session 5 ITR (SD; bits/min)	11.36 (6.53)	5.79 (2.61)

### Questionnaires

The average NASA-TLX workload score did not change across sessions (Friedman ANOVA, *χ*^2^_(4, *N* = 15)_ = 3.22, *p* = 0.52). The global average score was 63.2 (across sessions and participants; *SD* = 16.3). The mean scores of the NASA-TLX are summarized in Table 7. Descriptively, the weighted averages of the dimensions “mental demands” (*M* = 20.2) and “effort” (*M* = 17.5) contributed most to total workload scores. Conversely, “physical demands” (*M* = 3.2) and “temporal demands” (*M* = 4.0) were rated low. Bonferroni-Holm corrected Wilcoxon tests for each session revealed that mental demands were rated significantly higher than physical demands (all *p* < 0.001). Many participants proactively reported that ignoring non-target vibrations was very difficult and that they had perceived a high mental strain when using the system.

**Table 6:** Satisfaction ratings and average TUEBS scores.

Session	Satisfaction	TUEBS (main)	TUEBS (BCI)
1	6.8 (1–10)	4.5 (3–5)	4.3 (3–5)
2	7.3 (2–10)	-	-
3	6.7 (2–10)	-	-
4	7.1 (1–9)	-	-
5	8.0 (3–10)	4.2 (3–5)	4.2 (3–5)

*All values averaged over all participants. Parenthesis indicate ranges (rounded to the nearest integer)*.

**Table 7:** NASA-TLX scores.

Session	MD	PD	TD	P	E	F	Total	Raw
1	17.8	3.6	2.8	13.4	19.3	9.0	60.8	50.4
2	22.5	4.0	3.4	7.5	17.7	12.0	67.3	55.5
3	21.1	4.1	3.9	8.8	18.3	12.0	67.2	55.4
4	20.5	1.7	4.9	7.8	16.0	8.6	59.5	50.8
5	19.0	2.6	4.9	7.6	16.4	10.5	61.1	52.3
Mean	20.2	3.2	4.0	9.0	17.5	10.4	63.2	52.9

*MD = mental demands; PD, physical demands; TD, temporal demands; P, performance; E, effort; F, frustration; Total, global NASA-TLX score; Raw, unweighted global score. Value ranges are 0–100*.

Satisfaction, as measured with VAS and TUEBS, is shown in Table 6. The mean overall satisfaction with the BCI system increased from 6.8 (*SD* = 2.8) between the first session to 8.0 (*SD* = 2.2) and the last. There was a highly significant relationship between satisfaction ratings and ITR (*r*_s_ = 0.62, *p* < 0.01; Spearman’s Rho), but only when including BCI inefficient cases in the test.

The TUEBS score averages remained relatively stable between first and last sessions (*M*_1_ = 4.47 and *M*_5_ = 4.24; main items), indicating that participants felt “quite satisfied” (4) to “very satisfied” (5) with the system. This was also the case for the BCI specific items (*M*_1_ = 4.30 and *M*_5_ = 4.18). No significant effect of session number was found for either main or BCI specific items (paired *t*-tests, both *p* < 0.05).

We assumed that participants would be most competent to judge the BCI system at the end of the training, hence, we report the TUEBS from the fifth session, with a focus on the mean scores of the BCI specific items. Participants indicated they felt “quite satisfied” (mean score 4.2) with the systems’ Robustness/Reliability. In a comment, one participant speculated that electrode cables may break or fall off under stress. The systems Speed (the time required for a command) was also rated as “quite satisfactory” (3.9). Two participants reported that commands took “relatively long” or “too long,” another participant proverbially remarked that “practice makes perfect.” The BCI’s Learnability was again rated “quite satisfying” (4.0). One participant considered the BCI “effortful.” Two participants who did not reach BCI efficiency reported that “it probably requires a lot of practice” and that, despite some “habituation,” “there was no noticeable improvement.” Overall, the Aesthetic design of the technology was rated as “more or less satisfactory” (3.2), however, with several negative remarks concerning the EEG cap, cables, and gel (e.g., “gel in the hair, the cap is not pretty”).

One BCI specific TUEBS item inquired about which factors the participants considered most important for usability. After the fifth session, the factors Ease of use (*N* = 12), Learnability (*N* = 8), Efficiency (*N* = 6), and Robustness (*N* = 6) were mentioned most frequently (three factors could be selected).

## Discussion

We present a slightly adapted and extended replication of the study by Herweg et al. (2016). For the navigation task, the number of sequences was adjusted individually to an estimated minimum and thus precluded any ceiling effects as suggested by Herweg et al. (2016). Unfortunately, we found high discrepancies in the levels of BCI control between participants, with many of them not achieving BCI efficiency. For this reason, training effects were investigated for BCI efficient participants only.

### P300 Features

In visual paradigms, the P300 is usually strongest at parietal sites (Ravden and Polich, 1999). In the present study, however, amplitudes at position Pz were the lowest (see Table 2). The strongest deflections were observed at Cz, which is in line with several other publications with the tactile modality (Thurlings et al., 2012b; van der Waal et al., 2012; Kaufmann et al., 2014; Severens et al., 2014). This shift toward central sites may be a specific effect of tactile stimulation.

A review of recent literature from other P300 based BCI studies offers inconsistent findings of training effects on the (non-visual) P300: Some report increases with training (Halder et al., 2016; Herweg et al., 2016), while others suggest that motivation, not training, was a key factor (Baykara et al., 2016). In our case, the extracted ERP features mean amplitude and mean difference did not reveal a significant training effect, although a trend toward increasing P300 values could be seen at positions Fz and Cz. Our hypothesis (H1) about increasing physiological measures with training was thus not sufficiently supported by the present sample. For this analysis, the Friedman ANOVA (though appropriate) might not have been sensitive enough to reveal small effects.

Despite including only BCI efficient participants, peak P300 amplitudes after training (6.21 μV at Cz), were substantially lower than in the study by Herweg et al. (2016), who reported values after training of up to 9.2 μV (Fz). It should be noted that in both studies, the analysis of the P300 features was limited to certain electrodes in a fixed time window and was applied to the entire group indiscriminately. An analysis of machine-learning-based BCI performance, as discussed in the next section, should better account for interindividual differences.

### BCI Performance

Online ITRs during navigation increased highly significantly across the five training sessions, with mean values more than tripling between the first and last sessions (3.10–9.50 bits/min). Thus, we consider H2 (increasing ITR with training) confirmed.

This ITR mean value after training of the present study even exceeds the respective 4.98 bits/min from the study by Herweg et al. (2016) approximately by a factor of two. This is because Herweg et al. (2016) applied a fixed number of eight sequences, strictly limiting the ITR values that can be reached. The authors included an optional bonus task after the last session, in which this number of sequences was considerably reduced, and reported a mean ITR of 20.73 bits/min.

The highly significant increase of mean offline accuracies (based on calibration data using 10 sequences) with training further supports H2. Even without clear evidence from the analysis of physiological P300 features, this increase indicates that training affected brain activity patterns which in turn led to an improvement in classification. Importantly, it also suggests that the observed increase in online ITRs was not exclusively due to reductions in the number of sequences. In fact, despite our attempt to normalize online performance by adjusting the number of sequences, we found a significant increase in online accuracies with training (see Figure 5). Descriptively, this effect was strongest during the first three sessions, after which online accuracies appeared to consolidate. However, no other measure exhibited this pattern, rendering it difficult to interpret. It may be that for this wheelchair navigation task, the participants first had to become used to the novel 3D environment itself, which would be a factor only loosely related to the actual BCI system.

Overall, the significant increases in ITR and both online and offline accuracies across sessions strongly suggest that training had a positive effect on BCI performance. The substantial decrease of the required number of sequences indicates that this effect was caused not only by increases in online accuracy but also by a decrease in the time required for a command.

BCI Efficiency and Age Effects: Since our sample comprised six cases of BCI inefficiency, we must reject the hypothesis that all participants would reach more than 70% accuracy after training (H3). In the same vein, based on the encouraging results of the previous study (Herweg et al., 2016), we had hypothesized that advanced age would not be a limiting factor in BCI performance (H4). Yet, according to the present sample in which the younger group strongly outperformed the middle-aged group, this hypothesis was not supported. In fact, 57% of the middle-aged group did not reach BCI efficiency, in contrast to 25% of the younger group. In comparison, the percentage of inefficiency in visual BCIs in the population is estimated at only 20% (Allison and Neuper, 2010).

A possible explanation for this age discrepancy in performances could be that certain mechanoreceptors (e.g., Pacinian and Meißner corpuscles) decline in sensitivity or become less numerous with increasing age (Cauna, 1964; Iwasaki et al., 2003). Consequently, the middle-aged group may have been less sensitive to tactile stimulation. In a recent study, Chen et al. (2019) specifically compared the EEG responses to vibrotactile stimulation between older and younger samples. In line with our observations, the authors found several significant differences between the groups, most notably a decreased classification accuracy in the older sample. They attributed these findings primarily to the natural aging of the central nervous system, but also to changes in skull thickness and skin sensitivity.

On the other hand, mean amplitudes and overall performances of elderly participants in the study by Herweg et al. (2016) were much higher than even in our younger group, despite the exclusion of inefficient participants. We consider random sampling effects responsible for this inconsistency, as the paradigm itself was almost identical. Thus, we would still encourage the use of this tactile paradigm for elderly users because of its previously demonstrated feasibility among this population, and to elucidate further the potential performance of this age group.

### Workload, Satisfaction, and Usability

We included the NASA-TLX after every session to explore the perceived workload of the BCI operation. Scores did not significantly change over time. The mean global score was at a rather high value of 63.2, which falls into the 80% percentile according to a meta-study on the NASA-TLX (Grier, 2015). This score is slightly higher than those of similar BCI studies that also included the TLX, for instance, the 57.5 mean score from an auditory BCI by Käthner et al. (2013). However, the visual BCI from the same study was judged as much less workload intensive (*M* = 36.1). Another example is provided by Riccio et al. (2015), who reported a relatively high median score of 52.3 for a visual BCI. To conclude, the overall workload of our tactile paradigm seems to be on the higher end of the workload from comparable studies.

Analyzing the subscores of the NASA-TLX, we found that mental demands were rated much higher than physical demands, suggesting that operating the system provokes a high cognitive workload. It is unclear whether the paradigm itself can be simplified further, as it consists of only four classes that are positioned on the body in a way that necessitates no memorization of their meaning. Instead, the high mental demand might be inherent in the vibrotactile modality, which humans are less accustomed to using (as compared to the visual modality, for instance). It seems likely that ignoring non-target stimuli is much more difficult in the tactile paradigm than for example in a visual paradigm, in which users simply focus their gaze on the target only. Hence, gaze pre-filters the stimuli, making it comparatively easy to ignore non-targets. Such selective perception is not possible for tactile stimuli so that the filtering must be performed mostly on a cognitive level. Continued training in a consistent environment, however, might lead to automatization of the task (Logan, 1988) and alleviate some of the mental demands.

Mean satisfaction ratings measured with the VAS ranged between 6.7 (session 3) to 8.0 (session 5), indicating that participants had a mostly positive attitude toward the system. This result is similar to BCI studies that used the same scale: Mean ratings from Kübler et al. (2014) were 6.9–7.7, while another comparable study reported median values between 6.0–7.2 (Riccio et al., 2015). The strong correlation between satisfaction and performance (ITR) of the present study may indicate a negative influence of performance on the satisfaction ratings. However, some impaired end-users reported high satisfaction despite (subjectively) low performances (Holz et al., 2015).

Finally, mean scores of the TUEBS main items as well as of the extended BCI related items (both at *M* = 4.2 after session five) indicate high general satisfaction with the system. In a study by Kübler et al. (2014), four different applications were evaluated, with similar averages ranging between 3.7 and 4.2 for main items and 3.5 and 4.4 for BCI specific items. The TUEBS revealed no effect of training, possibly because the few days of participation were too limited to allow for substantial changes of opinion about the BCI system. The TUEBS however, revealed which factors participants considered most important for the usability of the system, illustrating above all that the “ease of use,” but also “learnability” was considered crucial.

## Conclusion

We demonstrated that virtual wheelchair control with the tactile BCI system can be feasible and that efficient control is achievable, albeit not by all participants, and that age likely contributes to inefficiency. Overall, BCI inefficiency may be more prevalent in tactile than in visual paradigms.

While physiological P300 measures increased only descriptively with training, we provide evidence for training effects on BCI performance measures (accuracy and ITR). On average, our participants did not reach the same high-performance level as in Herweg et al. (2016), possibly because of random sampling effects or a regression to the mean. Especially performances within our middle-aged group were in strong contrast to previous results. This should be further investigated, considering the often advanced age of potential end-users. A general interpretation, thus far, remains difficult, illustrating once more the need for designs that are individually tailored to the end-user. This necessitates identifying which factors are responsible for training-related improvements in BCI performance. These results were attained with healthy participants in a virtual environment—whether they are translatable to potential real-life use of powered wheelchairs by impaired patients remains to be explored.

## Data Availability Statement

The raw data supporting the conclusions of this article will be made available by the authors, without undue reservation, to any qualified researcher.

## Ethics Statement

The study was reviewed and approved by the ethical review board of the Institute of Psychology at the University of Würzburg, Germany. The participants provided their written informed consent to participate in this study.

## Author Contributions

ME and AK were responsible for experimental design and manuscript writing. ME was responsible for data acquisition and analysis. AK supervised the overall experimental design, data interpretation, and manuscript editing.

## Conflict of Interest

The authors declare that the research was conducted in the absence of any commercial or financial relationships that could be construed as a potential conflict of interest.
